# Effect of Fish Collagen Hydrolysates on Type I Collagen mRNA Levels of Human Dermal Fibroblast Culture

**DOI:** 10.3390/md16050144

**Published:** 2018-04-26

**Authors:** Ana Sanchez, Maria Blanco, Begoña Correa, Ricardo I. Perez-Martin, Carmen G. Sotelo

**Affiliations:** Instituto de Investigaciones Marinas (CSIC), Eduardo Cabello 6, 36208 Vigo, Spain; asanchez@iim.csic.es (A.S.); mblanco@iim.csic.es (M.B.); begonacorrea@iim.csic.es (B.C.); ricardo@iim.csic.es (R.I.P.-M.)

**Keywords:** fish collagen, collagen hydrolysates, fibroblast, type I collagen mRNA, commercial collagen hydrolysates

## Abstract

Fish discards and subproducts may represent an important source of raw material, not only for the food industry, but for other different kind of industries, such as the nutraceutical and cosmetic industries. Collagen, which is mainly obtained from animal skins, is an important structural protein in the animal kingdom having many different applications. It is well known that fish skins constitute a significant subproduct in the fishery industry, especially in the case of some species, where fish skins may represent up to 20% of the total body weight of fish. Peptides from collagen hydrolysates have been described to be useful for preventing skin aging and osteoarthritis, however, the mechanism for these biological activities is not well known. Fibroblasts are the main cell types involved in the collagen synthesis, and in the present work, human dermal fibroblasts have been exposed to the treatment of collagen peptides of two different molecular weight ranges. Results show that higher molecular weight collagen peptides produce higher synthesis of collagen type I mRNA and, therefore, it may suggest that prior molecular weight selection may be an important step to maximize the effect of collagen hydrolysates on collagen type I synthesis by dermal fibroblasts.

## 1. Introduction

Collagen is the main structural protein in connective, skin, and osteoarticular tissues of animal organisms [1]. The characteristic rope structure of collagen fibrils are organized by the union of basic units of collagen, formed by three subunit protein chains which are arranged as a triple helix and form large fibril stretches, which confer this protein its essential role in support tissues [2].

Collagen synthesis is essential for maintaining different body structures, such as skin, bones, and cartilage. Fibroblast cells are the main responsible for the synthesis of extracellular matrix (ECM) which contains, among other proteins, collagen [3]. 

Skin appearance depends on the collagen skeleton, for instance, wrinkle formation has been associated to decreased collagen synthesis and increased collagenase activity [4]. Dermal fibroblasts usually present a higher collagen synthesis rate than other tissues, since collagen plays an important role in the maintenance of skin integrity [3]. Some approaches to prevent or retard the apparition of wrinkles in humans are to use cosmetics or to intake nutritional supplements which help to maintain collagen molecules in the skin at optimum.

Some of these products include, as an active ingredient, collagen or hydrolyzed collagen [5]. Traditional sources of collagen for cosmetics are skins and hides from pigs and cows [6]. However, nowadays, collagen from marine origin is preferred, since it is free from animal species, causing religious concerns, and also the potential risks associated to the use of proteins from bovine subproducts, which may potentially be linked to mad cow disease [7,8,9]. 

Besides, sustainable socioeconomic and environmental principles promote the integral use of natural resources; this also applies to the fishery industry, which may represent an important source of valuable raw materials which are not always fully used. These raw materials, such as unused skins, are suitable for obtaining different products, such as collagen, gelatin, and collagen hydrolysates [10].

One of the main commercial species is *Prionace glauca* (Blue shark), which represents the sixth most important frozen fish species that landed in 2015 in the largest fishing port in Europe, located in Vigo (North-West Spain). A significant amount of this species is usually processed onshore and produces a substantial amount of skins (600 t/year, authors own data). The volume of fresh and frozen Blue shark traded in Vigo fish auction, accounted for up to 5568 t in 2015 [11]. The skin of these species is quite thick, especially in the case of mature females [12], and constitutes between 11.6% and 18% of total fish weight (not published data), making the skin one of the main targets for valorization of subproducts in this species. 

Skin subproducts of *Prionace glauca* have been used to obtain collagen and gelatin to produce films [13] and peptides with antioxidant capacity [14]. We have reported previously the production of collagen and collagen hydrolysates (CH) from *Prionace glauca* and other fish species, such as *Scyliorhinus canicula*, *Xiphias gladius*, and *Thunnus albacares*; we have tested antioxidative properties in the hydrolysates obtained [15], finding that similar antioxidative properties were found for *Prionace glauca* compared with other sharks. Collagen peptides with antioxidative, antihypertensive, or wound healing properties have also been obtained from different fish species, such as skate, Japanese flounder [16], Alaska pollack, squid [6], or Nile tilapia [17]. 

In the present study, human fibroblasts were cultivated in the presence of different amounts and molecular weights of Blue shark (*Prionace glauca*) skin collagen hydrolysates (PGLA-CH) and a commercial collagen hydrolysate (C-CH), with the aim of investigating the effect of these treatments on the collagen type I synthesis.

## 2. Results

### 2.1. Collagen Hydrolysates

PGLA pepsin soluble collagen (PSC) was used to obtain CH. As previously reported, PGLA PSC presented a characteristic type I Sodium Dodecyl Sulphate Polyacrylamide Gel Electrophoresis (SDS-PAGE) pattern with some bands of molecular weight lower than 116 kDa [18]. However, PSC exhibits an average lower molecular weight than the corresponding acid soluble collagen, although these low molecular weight peptides (<20 kDa) could not be seen in SDS-PAGE gels [15]. 

The PGLA PSC was further digested with alcalase, as previously described [15], for obtaining PGLA-CH, achieving a final hydrolysis degree of 16.52 ± 3.74%. This hydrolysate was freeze-dried and suspended in water, then ultrafiltrated, and two fractions were obtained: permeate fraction (PF), which represents the part of the PGLA-CH which went through the 3000 Da cut-off membrane; and the retained fraction (RF), which did not break through the membrane.

**Figure 1:**
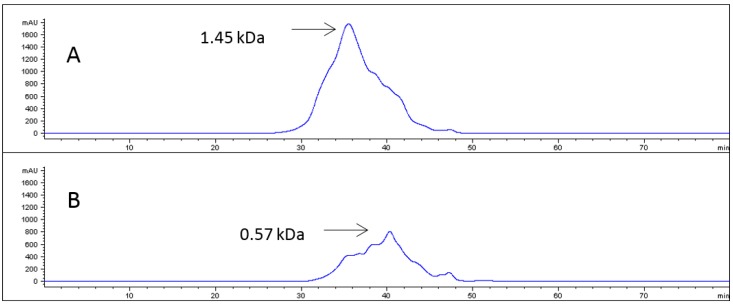
(**A**) Size exclusion chromatography profile of the alcalase hydrolyzed Blue shark (*Prionace glauca*) skin collagen hydrolysate (PGLA-CH) retained fraction (RF). (**B**) Size exclusion chromatography profile of the alcalase hydrolyzed PGLA-CH permeate fraction (PF).

Figure 1 shows the chromatographic profile of both PGLA-CH fractions and the average molecular weight obtained in each case. As can be observed in this figure, RF showed a peak of higher average molecular weight than PF, while the highest peak in RF corresponded to 1.45 kDa molecular weight, and the highest peak in PF is of about 0.57 kDa.

**Figure 2:**
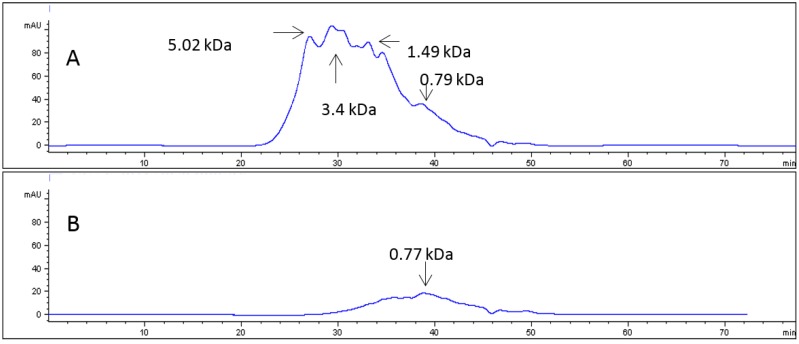
(**A**) Size exclusion chromatography profile of the commercial collagen hydrolysate (C-CH) RF; (**B**) Size exclusion chromatography profile of the C-CH PF.

Similarly, C-CH was also fractionated, and Figure 2 shows the chromatographic profile of the C-CH fractions, and as has been shown for PGLA-CH, the PF exhibited a lower average molecular weight than RF, however, in this case, both average molecular weights were higher than those obtained for PGLA-CH RF and PF.

Table 1 shows amino acid composition for the four hydrolyzed fractions, and it can be observed that the profiles were very similar, and only some slight differences in the main amino acids, such as glycine, alanine, proline, and hydroxyproline, were found.

Gly content was higher in C-CH PF and Hydroxyproline (HyPro)was higher in both RF fractions compared with PF. 

### 2.2. Effect of CH on mRNA Collagen Synthesis of Fibroblast Culture

**Figure 3:**
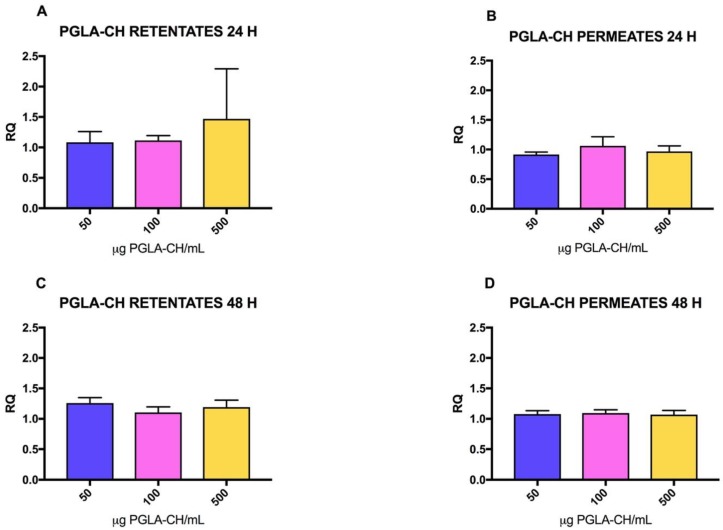
Relative quantification (RQ) values for collagen type I mRNA with increasing amounts of PGLA-CH (μg/mL) added to fibroblasts. RQ values are the means of five determinations and error bars are standard deviations. Blue bars: 50 μg/mL CH, Pink bars: 100 μg/mL CH, Yellow bars: 500 μg/mL. (**A**) RQ values obtained after 24 h treatment with PGLA-CH RF. (**B**) RQ values obtained after 24 h treatment with PGLA-CH PF. (**C**) RQ values obtained after 48 h treatment with PGLA-CH RF. (**D**) RQ values obtained after 48 h treatment with PGLA-CH PF.

The amount of PGLA-CH PF or RF added did not produce a significant increase in the collagen expression of the fibroblasts treated for 24 h with different concentrations of these extracts (*p* = 0.0959 and *p* = 0.4093, respectively). Furthermore, the treatment with the lowest concentration of PGLA-CH PF (50 μg/mL) showed relative quantification values (RQ values) with no overexpression or expression below the control; in the case of PGLA-CH RF, the highest concentration treatment (500 μg/mL) exhibited the highest RQ value, although not significant. There are no significant differences among RQ values after treating fibroblast with PF or RF after 24 h of treatment (*p* = 0.0735) with any concentration (50, 100, 500 μg/mL) (*p* = 0.3925), or in the interaction of both factors (*p* = 0.3534) (Figure 3).

In the case of the cells treated for 48 h with PGLA-CH fractions, a significant effect was found between the RQ value of cells treated with PGLA-CH RF or PGLA-CH PF (*p* = 0.0017). PGLA-CH RF produced higher RQ values than PF, as can be observed in Figure 3, however, in this case, RQ values regarding concentration (50, 100, 500 μg/mL) (*p* = 0.1879) or the interaction of both factors (*p* = 0.0813) were not significant (Figure 3). 

**Figure 4:**
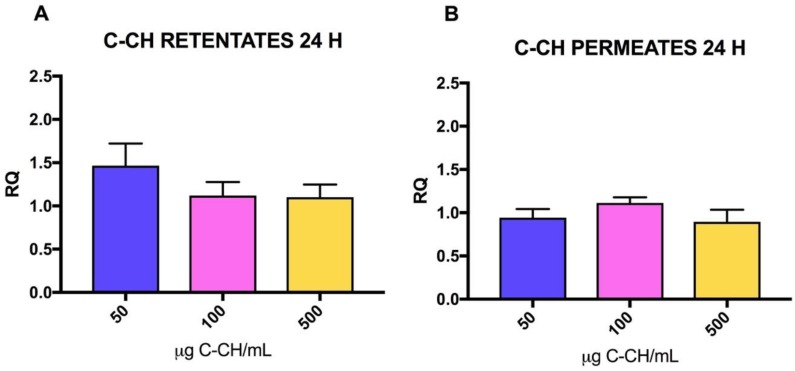
RQ values for collagen type I mRNA with increasing amounts of C-CH (μg/mL) added to fibroblasts. RQ values are the means of five determinations and error bars are standard deviations. Blue bars: 50 μg/mL CH, Pink bars: 100 μg/mL CH, Yellow bars: 500 μg/mL. (**A**) RQ values obtained after 24 h treatment with C-CH RF. (**B**) RQ values obtained after 24 h treatment with C-CH PF.

C-CH was also used to test the effect on the synthesis of mRNA collagen by fibroblast (Figure 4). Concentration did not produce significant effects in the RQ values after treatment with C-CH PF (*p* = 0.1106) during 24 h, while in the case of C-CH RF, significant differences were observed (*p* = 0.0181), in this case, a dose–response effect was observed with the lower concentration of C-CH RF (50 μg/mL) producing higher RQ values. Besides, as was found before with PGLA-CH fractions, significant differences were found between C-CH PF and RF RQ values (*p* = 0.0028), while neither concentration and interaction resulted in significance (*p* = 0.5918, *p* = 0.1245).

## 3. Discussion

Some previous studies had the objective of demonstrating that collagen hydrolysates can influence the collagen turnover/balance of different tissues, such as skin or cartilage, and that applying or ingesting collagen hydrolysates may be beneficial for preventing aging of skin or cartilages [19].

The wide range of molecular weight of peptides found in commercial bovine collagen hydrolysates indicates that different collagen sources, integrity, and enzymes may be used to produce those hydrolysates, calling into question the scientific basis of using collagen hydrolysates for improving collagen synthesis [20].

In this work we have used PSC from the skin of *Prionace glauca* and produced an alcalase hydrolysate which was characterized by HPLC, and this hydrolysate was compared with a commercial one, and it was found that the average molecular weight of both hydrolysates was different. The commercial hydrolysate does not declare the species from which the collagen was obtained, and it does not indicate how this hydrolysate was produced. Also, non-commercial hydrolysate contains magnesium (31.6 mg of magnesium salts and 600 mg of hydrolyzed collagen were present in C-CH). The main differences with PGLA-CH were the average higher molecular weight and the slightly lower Pro content of the C-CH PF. We have observed that molecular weight has an influence in collagen synthesis when analyzing the PGLA CH; RF has shown, in general, higher RQ values than PF, and this is particularly striking after 48 h. In the case of the C-CH, the observation was the same: we observed that C-CH RF produced higher RQ values than C-CH PF, and that C-CH RF also produced higher RQ values than PGLA-CH RF.

Therefore, we suggest that one important factor to explain RQ values differences after treatment with CH is the average molecular weight of the peptides used for treating the cells. We also observed that in the case of the C-CH, the presence of some component may be interfering with the synthesis of mRNA. Magnesium intake has been associated with the modulation of collagen synthesis and the improvement of bone density [21], however, in this case, the amount present in the C-CH might be too high for treating fibroblast directly, in the case of 500 and 100 μg/mL treatments, preventing the synthesis of collagen type I mRNA (12.5 μg and 2.50 μg of Mg, respectively). 

Some authors have suggested that the presence or content of some amino acids may increase fibroblast activity (migration, chemotaxis, and proliferation), in particular, the presence of Pro-HyPro and HyPro-Gly close to fibroblast cells has been suggested as responsible for this fibroblast activity [22]. These findings are in concordance with our results, in particular, with the higher HyPro content of PGLA-CH RF and C-CH RF compared with PF fractions, showing also a higher collagen type I mRNA after 48 h treatment.

## 4. Materials and Methods

### 4.1. Raw Material

Frozen skin from blue shark, *Prionace glauca* (PGLA), was provided by the fishery industry Lumar S.L. (A Pobra do Caramiñal, Spain) and stored at −20 °C until used. Residues of muscle attached to the skin were removed, and then the skin was cut into small pieces (0.5 × 0.5 cm^2^), mixed thoroughly, and kept frozen at −20 °C until needed.

Fish species was confirmed by DNA analysis from a portion of the muscle attached to the skin using a previously described method [23].

### 4.2. Extraction of Pepsin Soluble Collagen (PSC) from Skin

Extraction of collagen from PGLA skin was carried out at 4 °C as described in [15]: 200 g of skin were mixed with 3 L of 0.1 N NaOH, stirring for 24 h, changing the alkaline solution three times, then the alkaline solution was removed, and the skins were washed with cold distilled water until neutral pH. Collagen extraction was carried out with 0.5 M acetic acid containing 0.1% (w/v) pepsin (0.5 U/mg; Acros Organics, Janssen Pharmaceutical, Geel, Belgium) for 24 h (1:40 skin weight/acid volume). The suspension was then centrifuged at 6000× *g* for 20 min at 4 °C, the residue discarded, and the supernatant salted-out with NaCl at a final concentration of 2.0 M. The precipitated collagen was dissolved in 0.5 M acetic acid, and dialyzed for 3 days against cold distilled water, using 12,000 Da cut-off dialysis membranes and continuous stirring. Dialyzed PSC was freeze-dried and was kept at −20 °C until it was used for hydrolysis.

### 4.3. Enzymatic Hydrolysis of Collagen and Characterization of PSC Hydrolyzate

Enzymatic hydrolysis was performed as previously described in [15]. A commercial collagen hydrolysate (Collagen with Magnesium, Ana Maria Lajusticia, Barcelona, Spain) was used for comparison. The product label indicated that 600 mg of hydrolyzed collagen and 31.6 mg of magnesium salts were present in a pill. There was no indication in the label of species or origin.

#### 4.3.1. Fractionation of the Hydrolysate

Freeze-dried hydrolysates (4 g) were dissolved in distilled water (1%) and ultrafiltrated in two steps using ultrafiltration centrifugal devices (Amicon Ultra-15 Unit, Merck Millipore, Darmstadt, Germany) with molecular weight (MW) cut-off of 10 and 3 kDa. This process resulted in obtaining two fractions: retained fraction (RF), with peptides ranging from 10 to 3 kDa MW, and permeate fraction (PF), with peptides lower than 3 kDa MW. Both fractions were freeze-dried and kept at −20 °C until use.

#### 4.3.2. Size Exclusion Chromatography

The MW distribution of hydrolysates was estimated by gel filtration chromatography in an Agilent 1260 Infinity system (Agilent Technologies, Santa Clara, CA, USA), equipped with a Superdex Peptide 10/300 GL column, with 0.1% TFA in 30% acetonitrile as eluent. 

Freeze-dried RF and PF were dissolved in eluent (0.1% TFA in 30% acetonitrile) (0.5 g/mL) and filtered through a 0.22 µm filter. Samples of 20 µL were injected, eluted at 0.4 mL/min, and monitored at 220 nm. The column was calibrated with standard proteins (Sigma-Aldrich, Saint Louis, MS, USA): cytochrome C (12,400 Da), aprotinin (6500 Da), angiotensin II (1046 Da), leucine-enkephalin (555 Da), Val-Tyr-Val (379 Da) and Gly-Tyr (238 Da).

### 4.4. Cell Culture and Treatment with PSC Hydrolyzates

Adult human dermal fibroblasts (P10858 HDFa from Innoprot, Derio, Spain) were used. Cells were incubated with fibroblast medium (FM) from ScienCell (Carlsbad, CA, USA), containing basal medium, 2% of fetal bovine serum, 1% of fibroblast growth supplement, and 1% penicillin/streptomycin solution, at 95% humidity and 5% CO_2_, in a flask in a temperature regulated incubator. Cells (50,000) were transferred into each well of a 24 well-plate and further incubated for 24 h at 37 °C and 5% CO_2_. Then, FM was removed from the wells and substituted by a FM medium containing the different CH fractions as explained below. Five replicates (wells) of fibroblasts treated with each concentration and type of CH, and were incubated for 24 h and 48 h. Once these periods were finished, total RNA from each well was extracted with Cells-to-CT 1-Step Taqman Kit (Ambion, Waltham, USA). Briefly, FM containing CH media were removed, and 400 μL of cold phosphate buffer saline (PBS) were used for washing each well. After this, 100 μL of the lysis buffer, provided with the kit, was added to each well and mixed thoroughly with the cells, leaving the mixture to stand for 5 min at room temperature. Then, 10 μL of stop solution was added to each well, mixed, and further incubated for 2 min. After that time, the liquid from each well was collected and transferred to a 1.5 mL Eppendorf tube. Finally, each well was washed with an additional 100 μL of cold PBS, and the resulting liquid collected and mixed with that previously obtained.

Freeze-dried CH PF and RF were dissolved in FM (1 mg/mL) and filtered through PVDF 0.22 μm. From this stock, serial dilutions were prepared using FM as needed (Table 2). A final volume of 500 µl was added to each well to treat the fibroblasts.

Total extracted RNA was measured with Nanodrop (260 nm), and RNA concentrations adjusted to 35 ng/μL. These RNA extracts were then maintained at −20 °C until analysis by RT-PCR.

### 4.5. RT-PCR Analysis

Real time PCR was used to quantify by relative quantification (RQ) type I collagen mRNA (COL I) by employing the reference gene GAPDH.

Real time PCR assays were carried out in a 7500 Fast Real-Time PCR System equipment (Applied Biosystems, Waltham, MA, USA) using the primers and probes shown in Table 3 [24,25], and the Express One-Step Superscript^®^ qRT-PCR Kit (Invitrogen, Waltham, MA, USA).

RNA (70 ng) were used for the RQ of collagen type I mRNA. Retrotranscription was performed at 50 °C during 15 min; then 2 min at 95 °C, and 40 cycles at 95 °C for 15 s followed by 1 min at 60 °C were used as amplification conditions.

### 4.6. Statistical Analysis

Each experimental treatment was performed using five replicates per culture well for each treatment and control. The RQ data for treated fibroblasts were obtained with untreated fibroblast controls. RQ results were evaluated using one-way and two-way analysis of variance (ANOVA) and the Tukey’s multiple comparison test using the statistical software GraphPad Prism version 7.00 for Mac OS X, GraphPad Software, La Jolla, CA, USA, www.graphpad.com. Significance was set to *p* < 0.05.

## 5. Conclusions

Our study reveals the importance of scientific studies for supporting any nutraceutical effects of collagen hydrolysates. We have also found that not only the amino acid composition of the peptides may be important in the stimulation of the collagen type I mRNA by fibroblasts, as has been previously suggested, but also the molecular weight of the peptides is relevant for the mRNA production. Further studies are needed to confirm that both a particular molecular weight and amino acid composition are required to stimulate collagen type I mRNA in fibroblasts, in vitro and in vivo.

## Figures and Tables

**Table 1:** Amino acid composition of PGLA-CH RF, PGLA-CH PF, C-CH RF, and C-CH PF (residues/100 residues). Values are means of three determinations ± standard deviation.

Amino Acid	PGLA-CH RF	PGLA-CH PF	C-CH RF	C-CH PF
Aspartic acid	4.39 ± 0.02	3.43 ± 0.03	4.98 ± 0.01	3.37 ± 0.05
Threonine	2.19 ± 0.02	2.31 ± 0.02	2.18 ± 0.01	2.44 ± 0.03
Serine	4.09 ± 0.15	4.35 ± 0.13	3.57 ± 0.10	3.71 ± 0.01
Glutamic acid	7.18 ± 0.02	6.92 ± 0.04	7.61 ± 0.05	5.99 ± 0.19
Glycine	33.97 ± 0.06	34.20 ± 0.16	32.56 ± 0.02	38.90 ± 0.21
Alanine	11.40 ± 0.10	13.55 ± 0.10	11.57 ± 0.01	11.46 ± 0.01
Cysteine	0.20 ± 0.03	0.29 ± 0.04	0.23 ± 0.07	0.28 ± 0.01
Valine	1.81 ± 0.05	2.48 ± 0.05	2.53 ± 0.03	3.15 ± 0.02
Methionine	1.38 ± 0.01	1.87 ± 0.11	0.86 ± 0.00	1.13 ± 0.03
Isoleucine	1.92 ± 0.02	1.49 ± 0.03	1.11 ± 0.01	1.58 ± 0.02
Leucine	2.07 ± 0.04	2.63 ± 0.01	3.21 ± 0.01	4.44 ± 0.01
Tyrosine	0.00 ± 0.00	0.09 ± 0.12	0.65 ± 0.01	1.22 ± 0.13
Phenylalanine	1.50 ± 0.01	1.49 ± 0.03	1.73 ± 0.02	2.79 ± 0.07
Histidine	0.49 ± 0.00	0.57 ± 0.01	0.42 ± 0.01	0.53 ± 0.02
Lysine	2.61 ± 0.02	2.50 ± 0.06	3.05 ± 0.01	2.76 ± 0.04
Arginine	4.95± 0.01	5.31± 0.01	4.65 ± 0.04	4.73 ± 0.22
Hydroxyproline	8.29 ± 0.04	6.07 ± 0.11	8.62 ± 0.07	5.07 ± 0.40
Proline	11.56 ± 0.02	10.46 ± 0.02	10.49 ± 0.06	6.45 ± 0.09

**Table 2:** Concentration of CH PF and CH RF prepared with fibroblast medium (FM) and used to treat fibroblast cells.

Concentration				
Treatment Components	500 µg/mL	100 µg/mL	50 µg/mL	Control
CH PF and RF	250 µL	50 µL	25 µL	-
FM	250 µL	450 µL	475 µL	500 µL

**Table 3:** Sequence of primers and probes used for RQ of collagen type I.

Primer/Probe	Sequence 5′→3′
GAPDH-Forward	GGAAGCTCACTGGCATGGC
GAPDH-Reverse	TAGACGGCAGGTCAGGTCCA
GAPDH-Probe	VIC-CCCCACTGCCAACGTGTCAGTG-MGB
COL_I-Forward	ATGCCTGGTGAACGTGGT
COL_I-Reverse	AGGAGAGCCATCAGCACCT
COL_I-Probe	6-FAM-ACCAGCATCACCTCTGTC-MGB
